# Diagnostics of Primary Immunodeficiency Diseases: A Sequencing Capture Approach

**DOI:** 10.1371/journal.pone.0114901

**Published:** 2014-12-11

**Authors:** Lotte N. Moens, Elin Falk-Sörqvist, A. Charlotta Asplund, Ewa Bernatowska, C. I. Edvard Smith, Mats Nilsson

**Affiliations:** 1 Department of Immunology, Genetics and Pathology, Uppsala University, Uppsala, Sweden; 2 Clinical Research Center, Department of Laboratory Medicine, Karolinska Institutet, Karolinska University Hospital, Huddinge, Sweden; 3 Department of Clinical Immunology, Children's Memorial Health Institute, Warsaw, Poland; 4 Science for Life Laboratory, Department of Biochemistry and Biophysics, Stockholm University, Stockholm, Sweden; Institute of Immunology, Rikshospitalet, Norway

## Abstract

Primary Immunodeficiencies (PID) are genetically inherited disorders characterized by defects of the immune system, leading to increased susceptibility to infection. Due to the variety of clinical symptoms and the complexity of current diagnostic procedures, accurate diagnosis of PID is often difficult in daily clinical practice. Thanks to the advent of “next generation” sequencing technologies and target enrichment methods, the development of multiplex diagnostic assays is now possible. In this study, we applied a selector-based target enrichment assay to detect disease-causing mutations in 179 known PID genes. The usefulness of this assay for molecular diagnosis of PID was investigated by sequencing DNA from 33 patients, 18 of which had at least one known causal mutation at the onset of the experiment. We were able to identify the disease causing mutations in 60% of the investigated patients, indicating that the majority of PID cases could be resolved using a targeted sequencing approach. Causal mutations identified in the unknown patient samples were located in *STAT3*, *IGLL1*, *RNF168* and *PGM3*. Based on our results, we propose a stepwise approach for PID diagnostics, involving targeted resequencing, followed by whole transcriptome and/or whole genome sequencing if causative variants are not found in the targeted exons.

## Introduction

Primary Immunodeficiencies (PID) are a heterogeneous group comprising over 200 diseases caused by congenital defects of the immune system. These diseases are mainly characterized by severe recurrent infections, and can often be life-threatening. Early diagnosis and treatment of PID are therefore critical for reducing disease-associated morbidity and improving patient outcomes [1]–[3]. Yet, while the clinical phenotype and the molecular basis of an increasing number of immunological defects have been characterized over the last years, a timely and accurate diagnosis of PID remains difficult in daily practice. Current diagnostic procedures for PID are highly complex, and involve an array of specialized testing including lymphocyte proliferation and cytotoxicity assays, flow cytometry, measurement of serum immunoglobulin levels, neutrophil function tests and complement analyses [4]. Next, DNA sequencing or genotyping can be used in order to definitively establish a PID classification, and determine the optimal treatment strategy. The selection of candidate genes to screen is thereby guided by each patient's individual clinical and immunological characteristics. However, determining which genes (or specific mutations) to assess is not always evident; over 200 PID genes have currently been described [5], but the prevalence of most genetic defects is low [6]. Furthermore, mutations in different genes can result in similar phenotypes (locus heterogeneity), while mutations in different parts of the same gene can present with distinct phenotypes (allelic heterogeneity) – further complicating mutation detection in PID. At yet another level, the individual testing laboratories accredited for PID analyses normally only cover a small percent of all disease genes, often requiring the patient samples to be distributed to different labs.

Thanks to the emergence of high throughput “next generation” sequencing (NGS) technologies, the development and implementation of more comprehensive diagnostic assays – allowing the simultaneous analysis of multiple genes – now seems within reach.

With its ability to generate sequence reads for billions of DNA fragments in parallel, NGS offers an ideal platform for molecular diagnostics of PID and other multigenic disorders, enabling both sample and target multiplexing, at an affordable cost and within a reasonable time frame.

In a diagnostic setting, a targeted resequencing approach – where only specific genes or regions of interest are captured and sequenced – seems most appropriate; it provides focused, hypothesis-driven assay designs that are typically more readily analyzable than the more complex whole genome or whole exome data sets. This certainly applies for PID, where many of the causal genes have been identified, thus obviating the need for whole exome sequencing in most cases. A targeted sequencing approach also allows directing more sequencing capacity to a specific area, resulting in a higher coverage of the region of interest – along with a considerably lower cost per sample. In addition, the size of targeted assays is compatible with the capacity of the currently available bench top sequencers, such as Illumina's MiSeq, 454's GS Junior system and Ion Torrent's Personal Genome Machine, which, thanks to their flexible throughput and short run-time are very suitable for use in the clinic [7].

The usefulness of a targeted NGS approach in PID diagnostics was recently demonstrated by Nijman et al. [8], who used array-based and in-solution enrichment techniques combined with SOLiD sequencing for mutation screening in 170 PID genes. Using these methods, they were able to accurately detect point mutations and exonic deletions in 41 patients with known genetic diagnosis. In addition, the applied NGS methods enabled the identification of causal mutations in 4 out of 26 patients with undiagnosed PIDs.

In this study, we adopted a selector-based sequencing capture assay (S1 Figure) [9] together with Illumina sequencing, to identify disease-causing mutations in 179 known PID genes. The usefulness of this assay for molecular diagnosis of PID was investigated by sequencing DNA from 33 patients, 18 of which had at least one known causal mutation at the onset of the experiment.

## Materials and Methods

### Patient material

The patients with known mutations were earlier diagnosed either in Poland or in Sweden and were included in order to validate our high throughput sequencing approach as well as the data analysis. Mutations in several genes as well as different mutation types were included (Table 1).

**Table 1:** SNV, indel and CNV detection in patients with known causal mutation(s).

Individual ID	Gene	Mutation (transcript level | protein level)	Mutation detected by NGS?	Comment
*Patients with known single base or short indel mutations*				
519	*CD40LG*	c.120delA | p.A40fs	Yes	
520	*IL2RG*	c.315dupT | p.Y105fs	Yes	
521	*WAS*	c.G400A | p.A134T	Yes	
524	*FAS*	c.C817A | p.Q273K	Yes	
525	*FAS*	c.G14A | p.W5X	Yes	
526	*FAS*	c.G199T | p.E67X	Yes	
527	*ATM*	c.381delA	No	Not covered by design
	*ATM*	c.7010_7011delGT	Yes	
528	*ATM*	c.7630-2A>C	Yes	
	*ATM*	c.T6145G | Y2049D	No	Low read depth (<20)
529	*NCF1*	c.353_354 del TC	Yes	
	*NCF1*	c.75_76 del GT	Yes	
530	*RAB27A*	c.G259C | p.A87P	Yes	
531	*ELANE*	c.597+5G>A	No	Low read depth (<20)
532	*ELANE*	c.C416T | p.P139L	No	Low read depth (<20)
533	*RAG2*	c.C913G | p.P305A	Yes	
	*RAG2*	c.T1357A | p.W453R	Yes	
534	*RAG1*	c.256_257delAA | p.K86fs119X	Yes	
535	*MVK*	c.C829T | p.R277C	Yes	
	*MVK*	c.C1162T | p.R388X	Yes	
536	*CXCR4*	c.1016_1017delCT | p.S339fs342X	Yes	
537	*BTK*	c.240+108 T/G	Yes	
*Patients with known CNVs*				
523	*XIAP*	c.1-32_877	Yes	
534	*RAG1*	c.2619del67 | p.P874fs956X	Yes	

Abbreviations: NGS: Next Generation Sequencing; CNV: Copy Number Variant

The patients with unknown mutations included in this study represented three different categories: (*i*) patients where the suspected causal gene was not included in routine PID diagnostic assays performed at the Clinical Research Center (Huddinge, Sweden), (*ii*) patients with a phenotype of agammaglobulinemia but where no *BTK* mutation had been found, and (*iii*) cases without any knowledge of which PID gene was causing the phenotype.

All research involving patient materials was approved and written consent was waived by the regional ethical committee in Stockholm, as detailed in Dnr 144/01.

### DNA and RNA extraction and cDNA synthesis

DNA was extracted from whole blood using FlexiGene DNA kit (Qiagen GMbH, Hilden, Germany) according to the manufacturer's protocol. For some patients whole blood was also collected in Paxgene Blood RNA tubes (PreAnalytiX GmbH, Hombrechtikon, Switzerland), which allows the storage of blood samples at room temperature for up to 72 hours without any degradation of RNA. RNA was then extracted by using Paxgene Blood RNA Kit (PreAnalytiX GmbH, Hombrechtikon, Switzerland) according to the manufacturer's instructions. 500 ng of total RNA was used for cDNA synthesis, with random primers, by using First-strand cDNA synthesis kit for RT-PCR (AMV) (Roche Applied Science, Mannheim, Germany) according to the manufacturer's recommendations.

### Target enrichment and high throughput sequencing

Selector assays were designed to cover all exons and UTRs +/- 25 bp of 179 PID related genes (selected based on the 2009 IUIS PID classification, as well as reports on additional genes presented at the 2010 European Society for Immunodeficiencies (ESID) meeting). For eight of these genes, also intronic sequences were included (S1 Table). Design was done using an *in house*-developed software based on the operating principles of PieceMaker [10] and Disperse [11]. The probes were done with the ProbeMaker software as described in Stenberg et al., 2005 [12]. In summary, a subset of 12702 fragments and corresponding selector probes were selected based on fragment length (100–600 nt), GC-content (20–70%), and aiming at double-probe redundancy over targeted regions, while avoiding repetitive genomic elements in the ends. The design covered 717783 bp (corresponding to 98.6% of the targeted region) and the missing bases were found to be in or near repetitive elements.

Detailed information on design coverage for the individual genes is provided in S1 Table.

The selector probes and target enrichment kits were ordered from Halo Genomics, and selection experiments were performed following the corresponding protocol.

High throughput Illumina sequencing was performed at GATC Biotech (Konstanz, Germany) using the standard sample preparation and sequencing protocols. The barcoded samples were mixed in an equimolar ratio and 76 bp single reads (on average 9.6±5.1 million reads per sample) were generated in 2 lanes on the GAIIx (Illumina, San Diego, CA, USA).

### Data analysis

All samples were aligned to the targeted regions (extracted from hg18, March 2006 assembly) using MosaikAligner version 2.1.33 (http://code.google.com/p/mosaik-aligner/) with the following parameters: -hs 15, -act 35, -mhp 100, -ms 7, -p 8, -minp 0.95 -mmp 0.05. These settings allow for a maximum of 5% mismatches in a read. To detect SNVs an in-house developed software was used, which only considers uniquely mapped reads with an alignment quality of at least 5 and read bases with a minimum base quality of 20. A SNV was set to be detected if the read depth was at least 20 and the variant was found in minimum 20% of the reads. To exclude false positive SNVs emerging from the method all detected SNVs appearing in more than three unrelateed samples were removed. The remaining SNVs were annotated using Annovar version 2012.03.08 [13].

Copy number alterations were detected by analyzing differences in sequencing depth at each targeted position having at least 20x sequencing depth in all samples. For each patient, copy number differences were quantified by computing the log2 ratio of the observed normalized number of reads at each position, divided by the average number of reads in the reference samples, at the same position. To avoid differences due to inter-experimental variations, only those samples run in the same enrichment experiment as the sample under analysis were used as reference samples.

### Sanger Sequencing

Selected target fragments (prioritized based on phenotypic information and NGS data) were amplified from genomic DNA and cDNA, when available, using a standard PCR protocol. Primer sequences are available upon request. Amplicons were examined by applying 2.5 µl of each PCR reaction on a 1.3% agarose gel. PCR products were purified using ExoSAP-IT (USB, Cleveland, Ohio, USA) according to the protocol and then sequenced with the ABI PRISM BigDye Terminator v1.1 cycle sequencing kit (Applied Biosystems, Foster City, CA, USA) using the PCR primers as sequencing primers. The sequencing was performed on an ABI 3730 or ABI 3130xl Prism DNA Analyzers, and data analyzed with DNA Sequencing Analysis software, version 5.2 (Applied Biosystems) and Sequencher version 4.7 (Gene Codes Corporation, Ann Arbor, USA).

## Results

### Target enrichment performance

Enrichment and sequencing reactions were carried out for 33 patients and 1 HapMap sample. The number of reads, read depth, coverage and enrichment specificity values obtained for each sample are listed in Table 2.

**Table 2:** Number of reads, average read depth, coverage and specificity values per. patient.

Sample Nr.	# Reads	Av. Read depth^a^	F_1x_ b	Fc_20x_ c	Specificity
519	12202280	1475	96,4%	92,3%	82,9%
520	12229538	1614	96,9%	93,0%	85,7%
521	21263470	2766	96,9%	94,6%	89,8%
523	4027895	1126	92,5%	90,3%	87,6%
524	13723120	1849	93,0%	79,8%	95,1%
525	11454814	1519	97,1%	92,2%	88,8%
526	5322124	702	90,1%	67,7%	96,0%
527	4027895	550	95,4%	84,6%	87,6%
528	5434832	733	95,3%	87,5%	87,6%
529	5067209	769	96,6%	88,4%	95,7%
530	4420856	616	96,2%	86,2%	91,1%
531	4234930	611	96,1%	86,4%	95,3%
532	5466803	809	96,2%	85,6%	95,7%
533	4571127	658	93,2%	75,9%	95,7%
534	4885406	672	96,1%	87,3%	90,3%
535	3720105	501	95,2%	85,1%	89,9%
536	6441019	857	94,6%	88,1%	87,0%
537	3934397	481	72,6%	65,4%	68,8%
538	10747913	1377	95,3%	91,3%	87,1%
539	7025899	987	96,5%	89,1%	91,7%
540	10282901	1458	96,2%	91,0%	92,8%
541	11356264	1533	96,1%	91,7%	90,8%
542	9284871	1234	95,8%	90,1%	90,2%
543	13272010	1792	94,7%	92,7%	90,7%
546	15594003	2105	97,1%	93,7%	89,8%
547	16861385	2002	93,0%	85,0%	89,7%
548	13679360	1865	96,2%	91,8%	86,2%
550	19197873	2571	97,2%	94,1%	88,6%
554	3362288	438	95,5%	84,2%	85,7%
555	5271352	714	96,4%	88,0%	89,2%
557	16197112	2143	96,8%	91,8%	91,3%
559	14830725	2323	96,9%	92,1%	93,6%
560	12263230	1904	96,9%	90,5%	94,1%
HapMap NA 12801	11560956	1581	97,6%	93,4%	91,7%

a Av. Read depth is the average number of reads obtained per position in the targeted region.

b F_1x_ =  fraction of targeted region covered by at least 1 read.

c Fc_20x_  =  fraction of captured fragments covered by at least 20 reads.

Overall, 95% of the targeted bases were covered at least 1x (Table 2, F_1x_). Of the captured bases, an average of 88% was represented by at least 20 reads (Table 2, Fc_20x_). The average read depth in the targeted region was 1304±662 reads per base.

Despite the high overall target region coverage, one gene (*CFD*) had an average read depth of less than 20 (over all samples). This gene was also reported to have a low coverage by Nijman and coworkers [8]. In addition, 38 genes (21%) contained at least one exon with an average read depth below 20.

### Optimization of variant filtering criteria: detection of known single nucleotide variants (SNVs) and short indels

In order to narrow down the number of potential disease-causing mutations per patient, identified variants were filtered according to the following criteria:

*Minimal read depth*. Only variants with a total read depth (*i.e.* variant + wt allele depth) of at least 20 are retained
*Minimal variant allele ratio*. Only variants with a variant allele ratio higher than or equal to 0.25 are retained
*Variant consequence*. Only stop codon-introducing (nonsense), missense, splice site-disrupting SNVs and insertion/deletion (indel) variants predicted to disrupt a transcript's reading frame are retained.
*b SNP annotation*. Heterozygous variants having an rs number are retained only if they are annotated as having clinical significance. Homozygous variants are retained irrespective of their annotation.

In addition, all variants that were present in more than three unrelated individuals were filtered away.

This filtering step allowed us to reduce the initial number of detected variants from an average of 4851±877 to 40±62. This number of potential disease-causing variants could be further reduced when taking phenotypic information into account (see further). However, in this first optimization stage, mutation analyses were performed irrespective of detailed knowledge of patient symptoms or signs – *i.e.* no phenotype based gene prioritization or filtering was applied.

For each patient, a list of potential disease causing mutations was prepared based on the filtering criteria described above. For patients with known mutations, the resulting lists were interrogated for the presence of these variants. At this stage, 17 out of 22 (∼77%) known single base or short indel variants were detected (Table 1). One of the missed mutations (*ATM* c.381delA) was not covered by design, and could therefore not be detected by our assay. Three other mutations (*ELANE* c.C416T and c.597+5G>A, and *ATM* c.T6145G) showed a low read depth at the variant position. The fifth missed variant (*NCF1* c.75_76 del GT) is a common mutation, caused by recombination of the NCF1 gene with its two pseudogenes, which have a delGT compared to the NCF1 sequence. This mutation was missed in our initial filtered data set because the variant allele, due to its presence at the pseudogene position, is found in all individuals, and hence filtered away. Allowance for this specific position during the variant filtering process enabled to also retain this variant in our final mutation list.

These final filtering conditions allowed for a detection of 18 out of 22 (∼82%) known single base or short indel variants, resolving the mutation status for 12 out of 16 individuals (75%). These conditions were applied further on, for the analysis of individuals with unknown mutations.

### Detection of known Copy Number Variants (CNVs)

**Figure 1:**
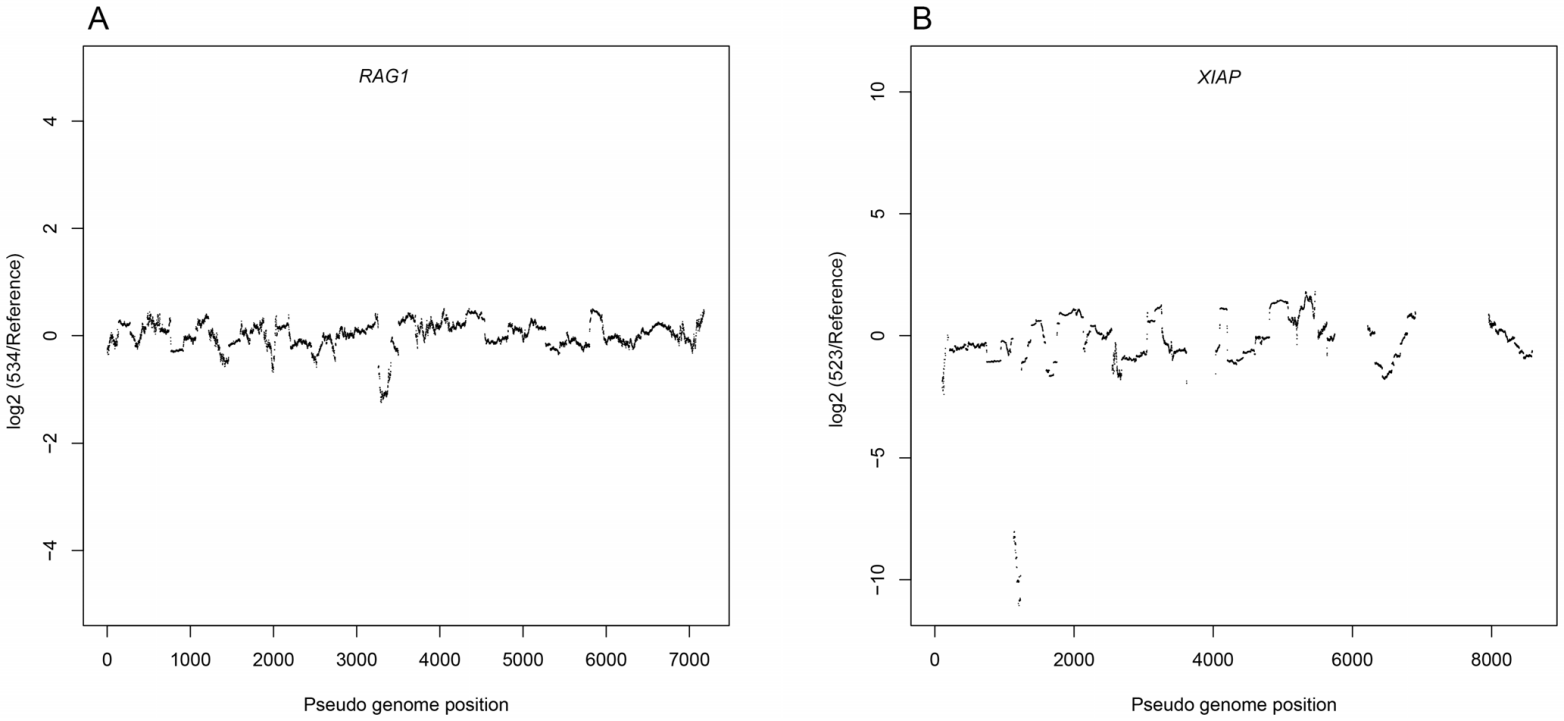
CNV plot showing detected copy number alterations in *RAG1* (patient 534, panel A) and *XIAP* (patient 523, panel B).

Next to SNVs and small indels, some types of PID may also be caused by structural variation. Therefore, CNV analyses were carried out for each of the sequenced samples. Two individuals had a known exonic CNV, the first one being a deletion of the complete exon 3 in XIAP (chr X: 122847162-122848070), and the second one involving a smaller deletion of 67 bp in RAG1 (chr 11: del36554050-36554116) (Table 1). We were able to detect both of these deletions (Fig. 1).

Including these CNVs, a total of 20 out of 24 (∼83%) known mutations were resolved by our assay, corresponding to the mutation status of 14 out of 18 individuals (∼78%).

### Mutation detection in patients with unknown disease causing variant

For fifteen patients in our sample the disease causing mutations were not known at the onset of the study. In order to identify potential causal mutations, SNVs and indels detected by NGS were first filtered as described above, resulting in an average of 16.5 mutations per patient. These lists of variants was then further prioritized based on phenotypic information, and subjected to Sanger sequencing. For 9 patients, no variants corresponding to the disease phenotype were found in the NGS data. In these cases, a single random variant was selected for validation.

Of the 22 mutations tested, 16 (72.7%) could be confirmed by Sanger sequencing. Of these 16 variants, 7 were considered potentially disease causing, based on the patients' phenotype. For 9 individuals, no potential causing SNVs or indels were found in the NGS data. A summary of the confirmed variants is given in Table 3.

**Table 3:** Mutations detected in patients with unknown disease causing variant.

Individual ID	Gene	Mutation (transcript level, protein level) a	Variant allele ratio in NGS	Mutation validated by Sanger?	Zygosity	Inheritance
*Patients with unknown mutations*						
EGS538	*STAT3*	**c.A2131G, p.I711V**	0,49	Yes	Heterozygous	Autosomal dominant
EGS539	*IGLL1*	**c.258delG, p.G86fs**	0,75	Yes	Homozygous	Autosomal recessive
EGS540	*IGLL1*	**c.258delG, p.G86fs**	0,85	Yes	Homozygous	Autosomal recessive
EGS541^b^^,^ c	*FERMT3*	c.G130A: p.G44R	0,29	Yes	Heterozygous	Autosomal recessive
EGS542	*PGM3*	**T>C, missense mutation** d	0,99	Yes	Homozygous	Autosomal recessive
EGS543^c^	*BLNK*	c.T923C, p.I308T	0,57	Yes	Heterozygous	Autosomal recessive
EGS546^c^	*NCF2*	c.C1183T, p.R395W	0,57	Yes	Heterozygous	Autosomal recessive
EGS547^c^	*IGLL1*	c.C443T: p.T148I	0,40	Yes	Heterozygous	Autosomal recessive
EGS548^c^	*IGLL1*	c.G560A, p.R187K	0,26	No	-	-
	*IGLL1*	c.G566A, p.R189H	0,26	No	-	-
	*TNFRSF13B*	c.706delG, p.E236fs	0,80	No	-	-
	*CD4*	c.G382A: p.V128M	0,47	Yes	Heterozygous	Autosomal recessive
EGS550	*STAT3*	**c.T1861A: p.F621I**	0,51	Yes	Heterozygous	Autosomal dominant
EGS554^c^	*UNC13D*	c.G2335A, p.V779M	0,61	Yes	Heterozygous	Autosomal recessive
	*UNC13D*	c.3010_3011insTG, p.P1034L	0,84	No	-	-
	*MPO*	c.C995T, p.A322V	0,51	Yes	Heterozygous	Autosomal recessive
EGS555^c^	*IGLL1*	c.C464T, p.P155L	0,34	No	-	-
	*IGLL1*	c.G475A, p.G159S	0,37	No	-	-
EGS557^c^	*IL12RB1*	c.C833T: p.T278M	0,53	Yes	Heterozygous	Autosomal recessive
EGS559^c^	*TICAM1*	c.G1702A: p.A568T	0,32	Yes	Heterozygous	Autosomal recessive
EGS560	*RNF168*	**A>G, missense mutation** d	0,44	Yes	Heterozygous	Autosomal recessive
	*RNF168*	**T>G, missense mutation** d	0,53	Yes	Heterozygous	Autosomal recessive

A clinical phenotype was reported as follows: EGS538 and 550 have symptoms and signs compatible with STAT3 mutations, including increased IgE-levels and *S. aureus* infections. Prior to the analysis EGS539 and 540 had 6% and <1% B-lymphocytes in peripheral blood and an increased susceptibility to bacterial infections. EGS542 has been prone to bacterial infections since childhood. EGS543, 546–548, 554, 555, 557 and 559 have reduced levels of B-lymphocytes and of immunoglobulins. EGS560 has a clinical phenotype related to common variable immunodeficiency, but also has congenital defects affecting non-lymphoid organs.

a (Potentially) causal mutations are listed in bold.

b Patient diagnosed with asplenia. Candidate genes not included in this assay.

c No potential causing variants were found for these individuals in the NGS data

d Manuscript in preparation.

In a next step, CNV analyses were performed for those patients where no causal SNV or indel could be identified. Four potential CNVs were detected based on our NGS data (located within intron 4 in *BTK*, intron 2 and the 3'UTR in *XIAP* and exon 16 in *BLNK*, respectively). None of these could be confirmed by further analyses (Sanger sequencing).

### Genotype-phenotype correlations for the identified mutations

Out of the unknown mutation samples, two (538 and 550) were found to harbor mutations in the *STAT3* gene. Mutations in this gene cause the ‘Hyper-IgE recurrent infection syndrome’, which is characterized by recurrent staphylococcal abscesses, pneumonias, extreme elevation of serum IgE, eosinophilia and connective tissue, skeleton and dentition abnormalities [14]. The disease is inherited as an autosomal dominant condition and is caused by the loss of function of 3 out of 4 dimers of the STAT-protein, resulting in reduced intracellular JAK-signaling. Both patients had phenotypes suggestive of this syndrome. Among the other patients, two sisters (539 and 540), were found to be homozygous for a mutation in the lambda 5 (*IGLL1*) gene, with their parents being heterozygous, healthy carriers. The *IGLL1* gene encodes a component of the pre-B-cell receptor, which is crucial for B-lymphocyte development [15].

One patient (560) inherited two different mutations in the *RNF168* gene, which encodes the E3 ligase RING finger 168. Only three patients with mutations in this gene have been reported to date and they suffer from radiosensitivity, immunodeficiency, dysmorphic features, and learning difficulties [16]. RNF168 has recently been reported to ubiquitylate 53BP1 and to control its response to DNA double-strand breaks [17]. Another patient (542) was found to carry a potentially disease-causing homozygous mutation in the *PGM3* gene. This gene was recently identified as the cause of a novel immunodeficiency by the use of a selector-based procedure [18] and whole exome sequencing [19], [20]. For both of these mutations we are currently studying the phenotypic consequences in greater detail and will report this separately.

## Discussion

Diagnosis of PIDs is highly complex due to substantial clinical, immunological and genetic heterogeneity. With the advent of next-generation sequencing, testing multiple genes in a single assay becomes possible, providing great opportunities for screening and diagnosing conditions with a heterogeneous genetic background.

In this study we have used a selector-based sequencing capture assay [9] to identify disease-causing mutations in 179 known PID genes. By sequencing 18 individuals with known mutations, we show that the developed sequence capture assay is capable of finding 83% of the causal variants, resolving the mutation status for 78% of individuals tested in one single assay. Since low read depth was shown to be the main reason for missing mutations (Table 1), the success rate could however be improved simply by increasing total read depth.

Out of our entire 179 gene panel, one gene (*CFD*) showed inadequate coverage (average read depth <20) upon sequencing. This gene was also reported by Nijman et al. to have a low overall coverage upon target enrichment and NGS, probably due to a high GC content and the presence of pseudogenes [8]. Apart from this gene, 21% of our target genes contained one or more exons with a low (<20) average read depth across all samples. An example is *ELANE* exon 4, where two mutations are missed using our NGS assay. This entire gene was found to be unanalyzable by Nijman et al. [8]. Comparable performance issues have been reported for other targeted NGS panels [8], [21], [22]. In a diagnostic setting, where obtaining sufficient sequence coverage is imperative for a reliable detection of genomic variants, poorly covered exons – especially those containing mutations hotspots – could be complemented by Sanger sequencing.

After sequencing and SNV detection, filtering of the obtained variants was needed in order to minimize the number of false positives and speed up the search for true mutations. Variant filtering criteria were rigorously optimized in order not to miss any mutations. When applying the optimized criteria on 15 patient samples with unknown mutations, an average of 16.5 variants were retained per sample, representing a manageable number for validation purposes. False positives were mainly due to the presence of pseudogenes, falsely aligning to the region of interest, and thereby creating artificial variants at the nonidentical positions. Sanger validation of a subset of these unknown variants resulted in 7 potentially disease causing mutations, distributed over 6 patients. For the remaining 9 patients, no variants corresponding to the disease phenotype were found in the NGS data. One of these patients was diagnosed with asplenia – for which no candidate genes were known at the time of this study. The other 8 individuals should be further investigated using RNA analysis of candidate genes, if required followed by whole transcriptome and/or whole genome sequencing.

Although our results illustrate the usefulness of the developed assay for detecting causal mutations in PID, the low resolution rate in patients with unknown mutations is obviously a major drawback. Indeed, less than half of the patients with unknown genetic diagnosis could be explained, a result that has been observed previously [8]. Since these patients were not selected for specific mutations or genes, the possible absence of these causal sequences in the assay is of course a logical explanation. The latest IUIS PID classification reports 240 causative genes [5], while only 179 were studied here. Furthermore, intronic sequences were not targeted (except for 8 X-linked genes), although several causal intronic mutations have been described in PID [6]. Also, we did not look for variants affecting splicing regulation (exonic or intronic splicing silencers and enhancers) in this study, even though the relevant regions were included in the design. Other possible reasons for the observed low resolution rate include the relatively low coverage by design, incomplete coverage upon sequencing, and difficulties with copy number detection. Though we were able to detect both of the known CNVs (in *RAG1* and *XIAP*, respectively), none of the 4 CNVs identified in NGS data in unknown patient samples could be confirmed by further analyses. This problem can, at least partially, be explained by the relatively short read lengths generated by the Illumina sequencer at the time of this study (76 bp single reads), which are difficult to align to the genome and may therefore hinder efficient copy number analyses. These alignment difficulties are even further complicated by the circular nature of the target fragments captured by the selector technology, which result in artificial junction reads (see below).

Various solutions are possible for increasing the resolution rate, the simplest being to just increase both read length and sequencing depth. In the present experiment, 88% of all captured bases were covered by at least 20 reads. Sequencing 20 times deeper would result in an average of 95% fraction covered. In a diagnostic setting – where one would only sequence a few samples per HiSeq lane or MiSeq run – this would certainly be achievable.

The most substantial improvement would however be gained by upgrading the assay design; in this study, a first generation of the HaloPlex target enrichment system (“selector method”) was used, amplifying the circularized captured fragments using multiple displacement amplification. This results in fragment libraries with random start points, and sequences encompassing artificial junctions formed by circularization of the targets (S1 Figure). Current HaloPlex assays however, use PCR in the final amplification step (S1 Figure) and therefore no longer result in artificial junctions, significantly facilitating post sequencing analyses (particularly CNV detection). Furthermore, both design coverage and capture efficiencies have been largely improved in the new HaloPlex system. Using a second generation HaloPlex design, 99.5% of the target region would be covered *in silico*, compared to 98.6% in the current assay. Combined with better sequence coverage and read depths – thanks to improved capture uniformity – this would greatly advance the assays' performance.

In this study, we have investigated the usefulness of a selector-based sequencing capture assay for identifying disease causing mutations in PID. Although the current assay clearly has some shortcomings, our results indicate that the majority of PID cases could be resolved using a targeted sequencing approach. With our current assay, we are able to explain 20 out of 33 (∼60%) investigated cases. Implementing the improvements described above, would most likely allow us to further increase this number. However, even with these assay modifications, completion with other methods will still be necessary.

We aimed to target the complete genes for 8 X-linked genes in this assay, while the rest of the genes were exon focused. While these genes made up ∼20% of our targeted sequence, we did not find any causative variants in these introns in the unknown patient samples, and we believe it is better to look for non-exonic variants by whole genome or transcriptome sequencing, if causative variants are not found in the targeted exons.

In general, we propose a stepwise approach for PID diagnostics: in a first stage, resolve most cases by a rapid and at low cost targeted enrichment assay, and then, in a second stage, follow up unexplained cases using whole transcriptome and whole genome sequencing.

Whole transcriptome sequencing is very useful for detecting alternatively spliced transcripts, which constitute an important class of mutations in PID. However, detection of genes with low expression levels is still difficult, and cell material from the correct tissue is not always available.

Whole genome sequencing is currently approaching the 1,000 USD milestone. Yet, the cost of a gene targeted library preparation kit is still much lower (∼100 USD per sample, and a sequencing cost in the same range). Current HaloPlex assays have a rapid turn-around time, and allow in principle enrichment and sequencing on a MiSeq instrument in one day. Further, the bioinformatic analyses and lists of potential mutation candidates generated in a targeted approach will be much shorter compared to whole genome sequencing, and involve less problems with potential incidental findings that might be difficult to make a decision whether to report or not [23].

Given these considerations, and based on our results, we believe that targeted sequencing is a preferred initial choice for PID diagnostics and has great potential as a first-line genetic test. The identification of genetic defects in patients with PID is however still a major challenge and implementing NGS in the medical laboratory will require robust quality assurance at multiple levels [24], [25]. Careful evaluation of the performance and “diagnostic yield” [24] will be essential to warrant the switch to NGS based analyses and ensure an effective translation of research to clinical practice.

## Supporting Information

S1 Figure **Selector workflow compared to the current HaloPlex Target Enrichment system.**
(DOCX)

S1 Table **Design coverage for the 179 target genes in the PID gene panel.**
(XLSX)

## References

[1] Bonilla FA, Bernstein IL, Khan DA, Ballas ZK, Chinen J, et al (2005) Practice parameter for the diagnosis and management of primary immunodeficiency. Ann Allergy Asthma Immunol 94:S1–63. 15945566 10.1016/s1081-1206(10)61142-8

[2] Oliveira JB, Fleisher TA (2010) Laboratory evaluation of primary immunodeficiencies. J Allergy Clin Immunol 125:S297–305. 20042230 10.1016/j.jaci.2009.08.043 PMC3412511

[3] Shehata N, Palda V, Bowen T, Haddad E, Issekutz TB, et al (2010) The use of immunoglobulin therapy for patients with primary immune deficiency: an evidence-based practice guideline. Transfus Med Rev 24 Suppl 1S28–50. 19962579 10.1016/j.tmrv.2009.09.011

[4] McCusker C, Warrington R (2011) Primary immunodeficiency. Allergy Asthma Clin Immunol 7 Suppl 1S11. 22165913 10.1186/1710-1492-7-S1-S11 PMC3245434

[5] Al-Herz W, Bousfiha A, Casanova JL, Chatila T, Conley ME, et al (2014) Primary immunodeficiency diseases: an update on the classification from the international union of immunological societies expert committee for primary immunodeficiency. Front Immunol 5:162. 24795713 10.3389/fimmu.2014.00162 PMC4001072

[6] Ochs HD, Smith CIE, Puck JM (2014) Primary immunodeficiency diseases. A molecular and genetic approach; Ochs HD, Smith CIE, Puck JM, editors. New York: Oxford University Press. 911 p.

[7] Desai AN, Jere A (2012) Next-generation sequencing: ready for the clinics? Clin Genet 81:503–510. 22375550 10.1111/j.1399-0004.2012.01865.x

[8] Nijman IJ, van Montfrans JM, Hoogstraat M, Boes ML, van de Corput L, et al (2014) Targeted next-generation sequencing: a novel diagnostic tool for primary immunodeficiencies. J Allergy Clin Immunol 133:529–534. 24139496 10.1016/j.jaci.2013.08.032

[9] Dahl F, Gullberg M, Stenberg J, Landegren U, Nilsson M (2005) Multiplex amplification enabled by selective circularization of large sets of genomic DNA fragments. Nucleic Acids Res. 2005/04/30 ed. pp. e71. 10.1093/nar/gni070 PMC1087789 15860768

[10] Stenberg J, Dahl F, Landegren U, Nilsson M (2005) PieceMaker: selection of DNA fragments for selector-guided multiplex amplification. Nucleic Acids Res 33:e72. 15860769 10.1093/nar/gni071 PMC1087790

[11] Stenberg J, Zhang M, Ji H (2009) Disperse—a software system for design of selector probes for exon resequencing applications. Bioinformatics 25:666–667. 19158162 10.1093/bioinformatics/btp001 PMC2647824

[12] Stenberg J, Nilsson M, Landegren U (2005) ProbeMaker: an extensible framework for design of sets of oligonucleotide probes. BMC Bioinformatics 6:229. 16171527 10.1186/1471-2105-6-229 PMC1239912

[13] Wang K, Li M, Hakonarson H (2010) ANNOVAR: functional annotation of genetic variants from high-throughput sequencing data. Nucleic Acids Res 38:e164. 20601685 10.1093/nar/gkq603 PMC2938201

[14] Freeman A, Grimbacher B, Engelhardt K, Holland S, Puck J (2014) Hyper-IgE recurrent infection syndromes. In: Ochs HD, Smith CIE, Puck JM, editors. Primary immunodeficiency diseases A molecular and genetic approach. New York: Oxford University Press. pp.489–500.

[15] Berglof A, Turunen JJ, Gissberg O, Bestas B, Blomberg KE, et al (2013) Agammaglobulinemia: causative mutations and their implications for novel therapies. Expert Rev Clin Immunol 9:1205–1221. 24215410 10.1586/1744666X.2013.850030

[16] Blundred RM, Stewart GS (2011) DNA double-strand break repair, immunodeficiency and the RIDDLE syndrome. Expert Rev Clin Immunol 7:169–185. 21426255 10.1586/eci.10.93

[17] Bohgaki M, Bohgaki T, El Ghamrasni S, Srikumar T, Maire G, et al (2013) RNF168 ubiquitylates 53BP1 and controls its response to DNA double-strand breaks. Proc Natl Acad Sci U S A 110:20982–20987. 24324146 10.1073/pnas.1320302111 PMC3876264

[18] Sassi A, Lazaroski S, Wu G, Haslam SM, Fliegauf M, et al (2014) Hypomorphic homozygous mutations in phosphoglucomutase 3 (PGM3) impair immunity and increase serum IgE levels. J Allergy Clin Immunol 133:1410–1419 e1413. 24698316 10.1016/j.jaci.2014.02.025 PMC4825677

[19] Zhang Y, Yu X, Ichikawa M, Lyons JJ, Datta S, et al (2014) Autosomal recessive phosphoglucomutase 3 (PGM3) mutations link glycosylation defects to atopy, immune deficiency, autoimmunity, and neurocognitive impairment. J Allergy Clin Immunol 133:1400–1405, 1400-1409, 1409 e1401-1405. 24589341 10.1016/j.jaci.2014.02.013 PMC4016982

[20] Stray-Pedersen A, Backe PH, Sorte HS, Morkrid L, Chokshi NY, et al (2014) PGM3 mutations cause a congenital disorder of glycosylation with severe immunodeficiency and skeletal dysplasia. Am J Hum Genet 95:96–107. 24931394 10.1016/j.ajhg.2014.05.007 PMC4085583

[21] Hoischen A, Gilissen C, Arts P, Wieskamp N, van der Vliet W, et al (2010) Massively parallel sequencing of ataxia genes after array-based enrichment. Hum Mutat 31:494–499. 20151403 10.1002/humu.21221

[22] Valencia CA, Ankala A, Rhodenizer D, Bhide S, Littlejohn MR, et al (2013) Comprehensive mutation analysis for congenital muscular dystrophy: a clinical PCR-based enrichment and next-generation sequencing panel. PLoS One 8:e53083. 23326386 10.1371/journal.pone.0053083 PMC3543442

[23] Evans BJ (2013) Minimizing liability risks under the ACMG recommendations for reporting incidental findings in clinical exome and genome sequencing. Genet Med 15:915–920. 24030435 10.1038/gim.2013.135 PMC3892767

[24] Weiss MM, Van der Zwaag B, Jongbloed JD, Vogel MJ, Bruggenwirth HT, et al (2013) Best practice guidelines for the use of next-generation sequencing applications in genome diagnostics: a national collaborative study of Dutch genome diagnostic laboratories. Hum Mutat 34:1313–1321. 23776008 10.1002/humu.22368

[25] Yu B (2014) Setting up next-generation sequencing in the medical laboratory. Methods Mol Biol 1168:195–206. 24870137 10.1007/978-1-4939-0847-9_11

