# Supplementary material for: Diagnostics of Primary Immunodeficiency Diseases: A Sequencing Capture Approach
Source: PLoS One. 2014 Dec 11;9(12):e114901. doi: 10.1371/journal.pone.0114901 (PMC4263707; doi:10.1371/journal.pone.0114901)
Supplement: S1 Figure — Selector workflow compared to the current HaloPlex Target Enrichment system. (DOCX) [file pone.0114901.s001.docx]

**
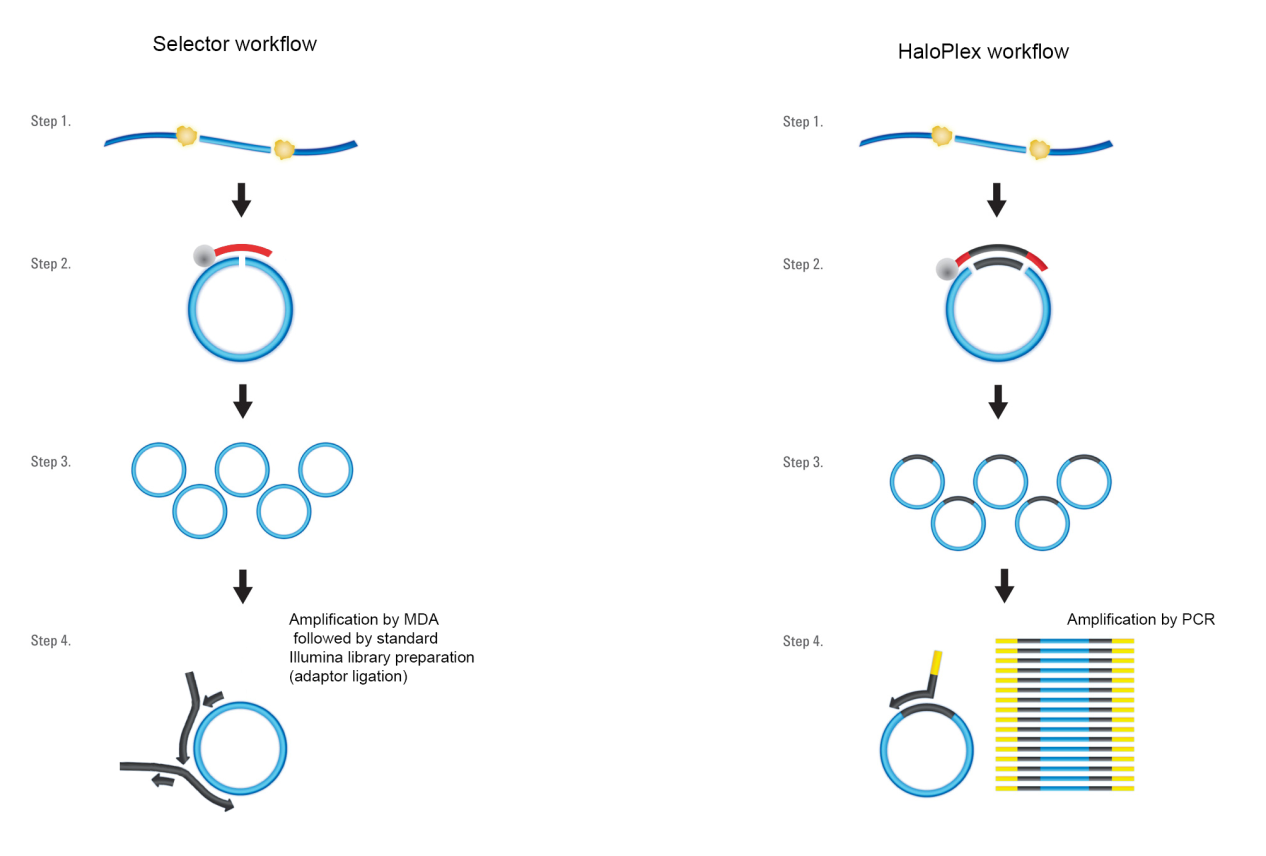
**

**Supplementary Figure S1:** Selector workflow (used in this study) compared to the current HaloPlex Target Enrichment system. The selector protocol creates artificial sequence junctions generated by circularization of the target fragments (without ligation of an external adaptor), followed by amplification by MDA. In the current HaloPlex protocol, these junctions are not formed. (Figure adapted from Agilent’s HaloPlex protocol)
